# Elevation of Nitric Oxide Synthase Activity by Dimethyladenosine from Silkworm Pupae in Aged Rats

**DOI:** 10.5487/TR.2008.24.3.169

**Published:** 2008-09-01

**Authors:** Mi Young Ahn, Jea Woong Han, Yoo Na Hong, Hwang Jae Sam

**Affiliations:** Department of Agricultural Biology, National Institute of Agricultural Science and Technology, 61 Seodun-dong, Kwonsun-gu, Suwon, 441-100 Korea

**Keywords:** Nitric oxide synthase, Silkworm, VEGF

## Abstract

This study examined the mechanisms underlying the effects of the vasorelaxation active substance (VAS), dimethyladenosine-5’-L-arabinose, and its partial purification fraction on nitric oxide synthase in improving erectile dysfunction with particular focus on the nitric oxide (NO)-cGMP pathways. Two rat models, 9-month-old SD rats and 11-month-old SD rats, were given VAS (40 mg/kg per day) for 4 days, The aqueous fraction of silworm male pupae extract; semi-purified VAS (100 mg/kg per day) for 10 days, respectively. The NOS activities of the following three enzymes were examined: neuronal NO synthase (*n*NOS), inducible NOS (*i*NOS), endothelial NOS (*e*NOS), vascular endothelial growth factor on endothelial cells (VEGF) and anti-inflammation effect of Tumor necrosis factor-α. The results showed increases in the nitric oxide synthase activities. Western blotting of the tissue homogenate showed an increase in the *n*NOS level in the brain and tongue, and an increase in the endothelial NO synthase (*e*NOS) level in penis. However, there was little association with VEGF production in HUVEC endothelial cells and no relationship with TNF-α which showed low levels.

## INTRODUCTION

Of the many crude insect drugs utilized in Oriental medicine, unmated silkworm male moths are used to treat erectile dysfunction to strengthen men's vitality. Silkworm larvae and pupae are currently registered as a food source, and 14 days after metamorphosis, the pupae can be substituted for the silkworm moth with the same efficacy (Ryu *et al.*, 2002). An ethanol extract of the pupae showed a tonic effect that increases the testosterone level in serum by 19% after a 3-week repeated treatment (Ryu *et al.*, 2002). Recently, dimethyladenosine-5'-L-arabinose was found to be the main component responsible for the improvement in erectile dysfunction, and may be a lead compound for the development and improvement of vasculogenic impotence drugs through phosphodiesterase inhibition and NO production in endothelial cells (Ahn *et al.*, 2008).

The mechanism of sildenafil citrate (Viagra) and other similar drugs for the treatment of erectile dysfunction is well known. When a man is sexually stimulated, either physically or psychologically, nitric oxide (NO) is released from non-cholinergic, non-adrenergic neurons in the penis, as well as from endothelial cells. NO production by cells requires the presence of one or more of the three isoforms of NO synthase (NOS) (Ziche and Morbidelli, 2000). Nitric oxide synthases (NOS) catalyze the production of NO and classified into neuronal NOS (*n*NOS), inducible NOS (*i*NOS) and endotheial NOS (*e*NOS) (Park *et al.*, 2004). NO diffuses into the cells, where it activates soluble guanylyl cyclase, an enzyme that converts GTP to cGMP (Fessenden *et al.*, 1998; Toda *et al.*, 2005). The cyclic nucleotide then stimulates protein kinase G (PKG), which initiates a protein phosphorylation cascade. This decreases the intracellular level of $Ca^{2+}$ ions, ultimately leading to a dilation of the arteries that transport blood to the penis and compression of the spongy corpus cavernosum. A phoshodiesterse (PDE) 5 inhibitor, which inhibits the enzymatic hydrolysis of cGMP in the human corpus cavernosum, can cause the same outcome (Kukreja *et al.*, 2004). NO significantly contributes to the prosurvial/proangiogenic program of the capillary endothelium by triggering and transducing cell growth and differentiation via endothelial-constitutive NO synthase activation, cyclic GMP elevation, mitogen activated kinase activation and fibroblast growth factor-2 expression (Ziche and Morbidelli, 2000). An increase of NOS activity in correlation with angiogenesis (Ziche and Morbidelli, 2000) and tumor necrosis factor-$\alpha$ (TNF-$\alpha$) was reported to produce during ischemia, and can induce vascular inflammation leading to endothelial dysfunction (Zhang *et al*., 2006).

This study examined the mechanisms underlying the effects of vasorelaxation active substance (VAS), a dimethyladenosine-5'-L-arabinose, and its partial purification fraction on the following: nitric oxide synthase in improving erectile dysfunction, the growth factor on endothelial cells (VEGF), and possible anti-inflammatory effect though the inhibition of of TNF-$\alpha$.

## MATERIALS AND METHODS

**Chemicals.** Dimethyladenosine and partial purificate of each purification step, from male silkworm (*Bombyx mori* L.) pupae supplied by the department of Agricultural Biology, National Institute of Agricultural Science and Technology, Korea. DEAE Sephadex A-25 purchased from Sigma Chemicals (St. Louis, MO).

**Cell culture and solutions.** Investment of NO production and cytotoxicity performed on calf pulmonary artery endothelial (CPAE) cells (ATCC CCL-209, Manassas, VA, USA) in Dulbecco's modified Eagle medium (GIBCO, New York, USA) supplemented with 10% newborn calf serum (GIBCO, New York, USA), 1 mM/l L-glutamine, 100 units/ml penicillin G and 100 μg/ml streptomycin sulfate (Sigma, St. Louis, MO) at 37°C in a humidified atmosphere of 5% $CO_2$ in air. Furthermore investment of NO production also performed on human umbilical vein endothelial cells (HUVEC) (ATCC, Manassas, VA, USA) in endothelial cell basal medium (EBM)-2 with EGM™-2 singlequots (Cambrex, Walkersville, USA) at 37°C, 5% $CO_2$ incubator.

The cytotoxicities of the purified fractions were tested against CPAE cell line using XTT {sodium 3'-[1-(phenylamino-carbonyl)-3,4-tetrazolium]-bis(4-methoxy-6-nitro) benzene sulfonic acid hydrate} kit solution (Boehringer Mannheim), as described previously (Geldof *et al*., 1999).

**Animals.** Specific pathogen free SD rats (male) normal 9-month-old ($n$ = 3, weighing 409.0 ± 7.0 g) and 11-month-old rats ($n$ = 5, weighing 655.5 ± 14.7 g), were purchased from Samtako Co. Ltd. (Osan, Korea), The rats, were housed in an environmentally-controlled room with 23 ± 1°C, a relative humidity of 55 ± 10%, air ventilation of 10~18 times/hr, a 12-hr light/dark cycle of 150~300 lux. The rats were fed with, a standard diet (Samtako Co. Ltd., Osan, Korea), and water *ad libitum* before the repeated-dose toxicity study testing began.

All procedures were carried out in accordance with the Korean Food and Drug Administration (KFDA) *Guidelines for Toxicity Tests of drugs and related Materials* (KFDA, 1999).

**Sample preparation having vasorelaxation activity.** The vasorelaxation substances (VAS) from the aqueous fraction were isolated by a combination of gel filtration and anion-exchange chromatography on DEAE Sephadex A-25 and reverse phase-HPLC. The active substance was identified to be a dimethyladenosine and dimethyladenosine-5'-L-arabinose, which have phosphodiesterase (PDE) inhibitory and NO production activity, as described previously (Ahn *et al*., 2007).

**Screening of expression level of *e*NOS, *n*NOS, *i*NOS in dot blot assay.** To analyze *n*NOS, *e*NOS, and *i*NOS, brain, tongue, testis and penile crural tissues were homogenized in liquid nitrogen and rehomogenized on ice in a 10-fold volume lysis buffer, PRO-PREP™ Protein extraction solution (iNtRON, Busan, Korea). Equal quantities (20 μg) of lysates are not separated electrophoretically but are spotted circular templates directly onto the PVDF membrane using Bio-Dot® microfiltration apparatus (Bio-Rad, New York, USA) as followed user's manual. Equal quantities (20 μg) of lysates were not separated electrophoretically but circular templates spotted directly onto the PVDF membrane using a Bio-Dot® microfiltration apparatus (Bio-Rad, New York, USA) according to the user's manual.

**Western blots.** Equal quantities (20 μg) of upper mentioned lysates and *n*NOS (~160 kDa), *e*NOS (~135 kDa), and *i*NOS (~130 kDa) standard (Cayman Chemical Company, MI, USA), were separated electrophoretically on 6~16% sodium dodecyl sulfate (SDS) gradient polyacrylamide gels (Invitrogen, Carlsbad, USA). Proteins were then electroblotted onto a PVDF membrane. The membranes were blocked, then incubated with polyclonal anti-*e*NOS antibody, anti-*n*NOS antibody and anti-*i*NOS antibody (Cayman Chemical Company, MI, USA) The chemiluminescence was detected using CDP-strar® Western blotting detection reagents (Invitrogen, Carlsbad, USA). The densitometric assessment of the bands on the autoradiogram was carried out using Vision work LS software (Seolin Bioscience,

Seoul, Korea).

**Endothelial *e*NOS assay by ELISA.** The levels of *e*NOS and VEGF production were measured from as antigen-antibody reaction using an enzyme-linked immunosorbantassay (ELISA) kit manufactured R&D systems (Minneapolis, USA), respectively. The cell homogenate from the brain, tongue, testis and penile crural tissues were examined using an *e*NOS kit.

**Calcium ion assay in sample treated rat.** Vasorelaxant substance such as sildenafil enhances platelet activation in the presence of subthreshold concentrations of thrombin or vWF (Li *et al*., 2003) and Nitric oxide (NO) is a potent inhibitor of platelet activation, that inhibits the agonist-induced increase in cytosolic Ca$^{(2+)}$ concentration through both cGMP-dependent and independent pathways (Crane *et al*., 2005). The levels of Calicium ion in sample treated rat tissues (brain, tongue, testis and penis) and sera were investigated using Calicium kit (Asan Pharm, Hwasung, Korea).

**Cytokine production measurements in splenocytes.** The levels of VEGF and TNF-α production were measured using an ELISA kit for TNF-α (Recombinant murine TNF-α kit and VEGF (mouse VEGF Immunoassay) purchased from QuantikineRM, R&D systems, Inc. (Minneapolis, MN, USA) according to the manufacturer's instructions.

The splenocytes were separated from a C57BL/6 female mouse (SLC Co, Shizuoka, Japan). The supernatants from the splenocytes incubated with dimethyladenosine-5'-L-arabinose and its partial purification fraction at 37°C were obtained at various time points, and the TNF-α and VEGF levels in these fractions were measured by ELISA.

**Endothelial VEGF assay.** The level of VEGF production was also measured in human umbilical vein endothelial cells (HUVEC) (ATCC, Manassas, VA, USA) in an endothelial cell basal medium (EBM)-2 with EGM$^{TM}$-2 singlequots (Cambrex, Walkersville, USA) at 37°C in an atmosphere containing 5% $CO_2$. The VEGF level was determined using a VEGF standard curve (Lee and Lee, 2005).

**Statistical analysis.** The results are presented as means ± standard deviations (S.D). The Student's *t*-test was used to establish the significances of differences between the control and treatment groups. $p < 0.05$ was considered statistically significant.

## RESULTS AND DISCUSSION

**Dot blot assay with *n*NOS, *e*NOS and *i*NOS antibody.** The level of neuronal nitric oxide synthase (*n*NOS) expression increased in brain and tongue by SWP purified fractions with dimethyladenosine-5'-L-arabinose (Fig. 1). In addition, there was an increase in the level of endothelial NO synthase (*e*NOS) and inducible nitric oxide synthase (*i*NOS) in penis and testis tissues homogenates, respectively (Fig. 1).

**Effects of dimethyladenosine on the expression of NO synthase in 9 month old rats.** The level of *n*NOS expression in the brain tissues of 9-month-old rats for 4 days was significantly lower than that of the normal rats ($p < 0.05$, Fig. 2). However, the expression levels were restored to ~58% of normal levels in aged rats after a treatment with dimethyladenosine ($p < 0.05$). Furthermore, the level of *e*NOS expression in the penises of the DMA treated and aged rats were also higher than that of normal rats, showing a 390.9% increase (Fig. 2).

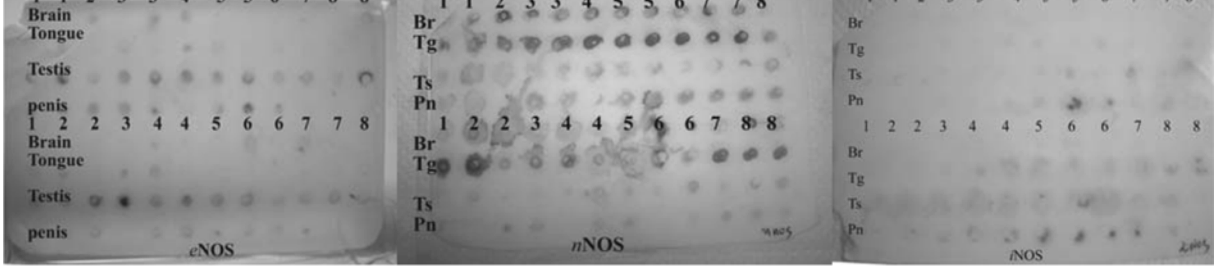

**Fig. 1.** The level of *e*NOS, *n*NOS, *i*NOS expression in the dot blot assay in various tissues of the SWP purificates-treated rat models. 1: Non treat; 2: saline; 3; SWP Aqueous fraction 100 mg/kg; 4: DX42-47: DEAE Sephadex A-25 fraction #42-47 pooling 40 mg/kg; 5:G10, 20-32: BioGel P10 #20-32 pooling 40 mg/kg; 6: G10, 14-19 BioGel P10 #14-19 pooling 15 mg/kg; 7: viagra 2 mg/kg; 8; IS aqua: *Isaria sinclairii* 100 mg/kg. Br; brain;Tg: tongue; Ts: testis; Pn: penis.

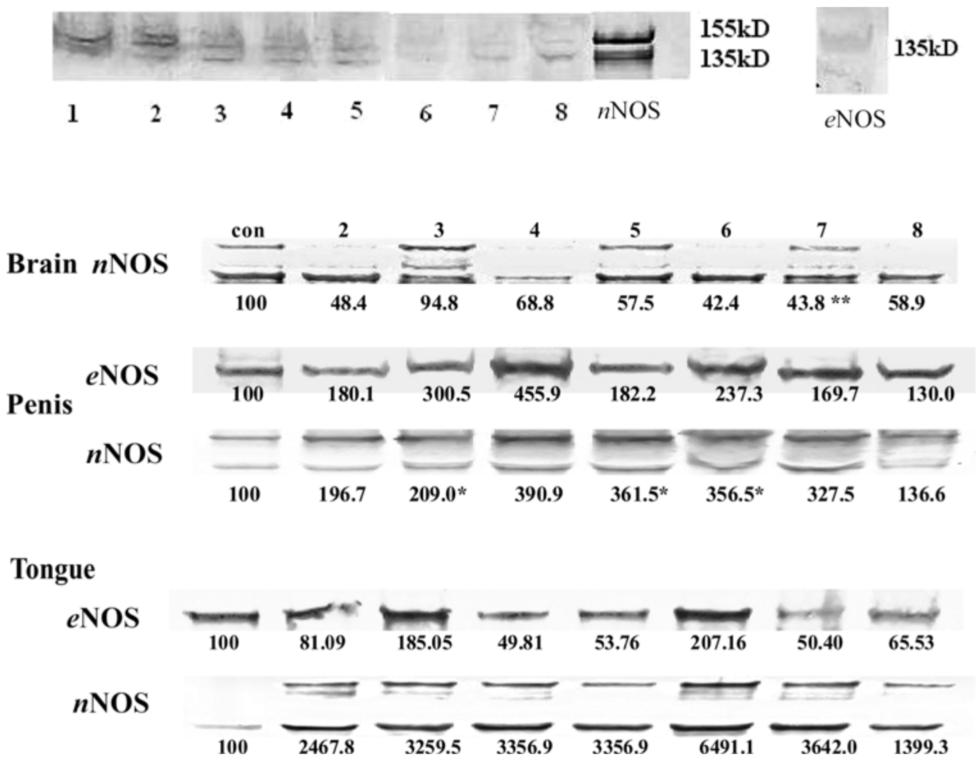

**Fig. 2.** Level of nitric oxide synthase expression in the western blots of various tissues of silkworm pupae purified substances-treated 9-month old rat models over a 4-day period. 1: Non treat; 2: saline; 3; SWP Aqueous fraction 100 mg/kg; 4: DX42-47: DEAE Sephadex A-25 fraction #42-47 pooling 40 mg/kg; 5:G10, 20-32: BioGel P10 #20-32 pooling 40 mg/kg; 6: G10, 14-19 BioGel P10 #14-19 pooling 15 mg/kg; 7: viagra 2 mg/kg; 8; IS aqua: *Isaria sinclairii* 100 mg/kg. Significantly different from the untreated controls (*$p < 0.05$).

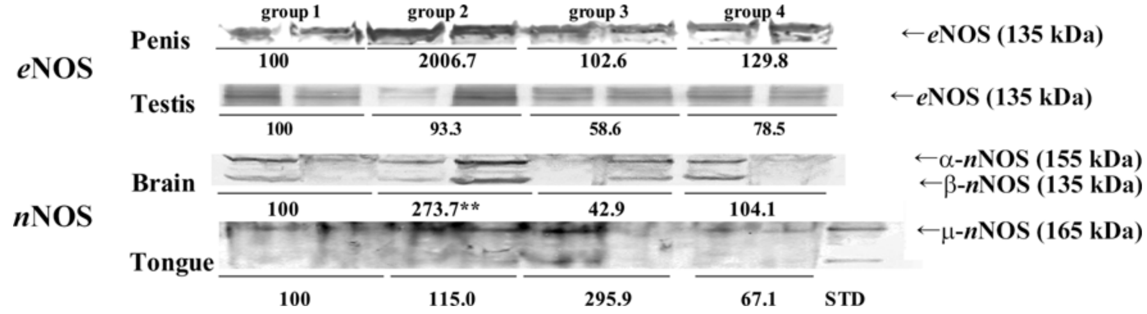

**Fig. 3.** Western blot analysis of nitric oxide synthase in various tissues of each aqueous fraction of the silkworm and *Isaria sinclairii* - treated 11-month old rat models over a 10-day period. I: PBS treat; II: silkworm pupae aqueous fraction 100 mg/kg; III: *Isaria sinclairii* aqueous fraction; IV: viagra 2 mg/kg, each. Significantly different from the untreated controls (**$p < 0.01$).

**Effects of dimethyladenosine on expression of NO synthase in 11-month-old rats with VAS (40 mg/kg per day) for 10 days.** The level of *e*NOS expression in the penile tissues of 11-month-old aged rats was significantly higher than that of the normal rats after treatment with the silkworm pupae aqueous (SWPA) fraction (Fig. 3). The expression levels after administering SWPA extract, *Isaria sinclairii* aqueous fraction and viagra was 206.7%, 102.6% and 129.8%. The level of *n*NOS expression in the brain tissues of the same aged rats treated with SWPA increased significantly showing a 273.7% increase compared with 104.1% for viagra. The specific target organ deposit could be related to brain: *n*NOS and penis: *e*NOS.

**Endothelial *e*NOS activity of dimethyladenosine.** As shown in Fig. 4, the *e*NOS activity in the brain tissues of the 11 month old rat model for 10 days

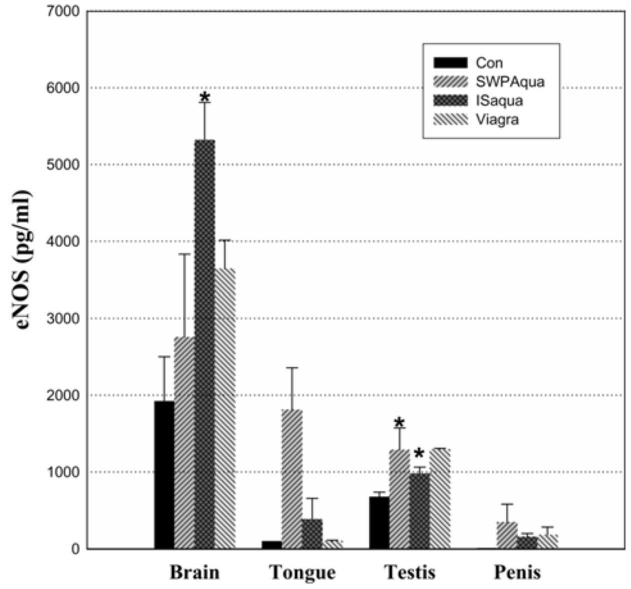

**Fig. 4.** Effects of the silkworm pupae aqueous fraction and *Isaria sinclairii* aqueous fraction on endothelial Nitric oxide synthase activity in the brain and testis tissues of 11-month aged rat models over a 10-day period. I: PBS treat; II: silkworm pupae aqueous fraction 100 mg/kg; III: *Isaria sinclairii* aqueous fraction; IV: viagra 2 mg/kg, each. Values represent means ± SE. Significantly different from the untreated controls (**p* < 0.05).

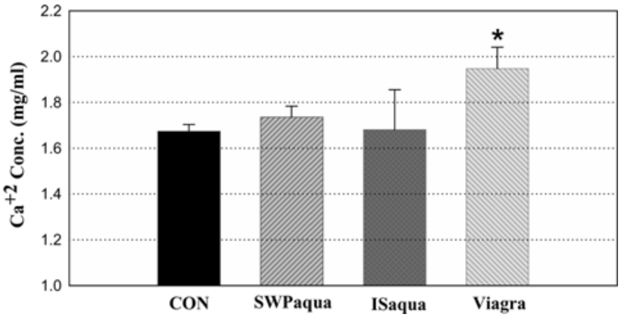

**Fig. 5.** Calcium ion concentration in the serum of the semi-purified fraction treated rat models over a 10-day period. I: PBS treat; II: silkworm pupae aqueous fraction 100 mg/kg; III: *Isaria sinclairii* aqueous fraction; IV: viagra 2 mg/kg, each. Values represent means ± SE. Significantly different from the untreated controls (**p* < 0.05).

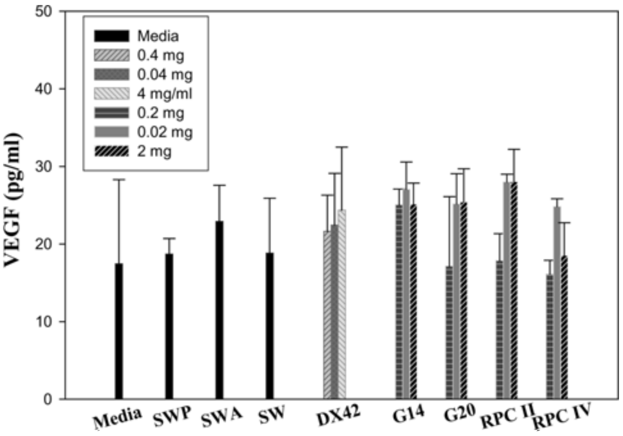

**Fig. 6.** Effects of the silkworm pupae extract on vascular endothelial growth factor (VEGF) in HUVEC cells. SWP: silkworm pupae ethanol extract 0.4 mg; DX: DX 2: DEAE Sephadex A-25 fraction V 0.04 mg; DX 20: DEAE Sephadex A-25 fraction V 0.4 mg; DMA: BioGel P10 gel filtration II with dimethyladenosine-arabinose 0.2 mg. Media; SNP: Sodium nitroprusside 100 μM; SWP: silkworm pupae ethanol extract 0.4 mg; SWA: silkworm male adult ethanol extract 0.4 mg; SW: silkworm ethanol extract 0.4 mg; DX 42-47, 0.4 mg, 0.04 mg, 4 μg, respectively;G10, 14-19; G10, 20-32; reverse phase chromatography (RPC) Fr II and IV with dimethyladenosine-arabinose, each 0.2 mg, 0.02 mg, 2 μg, respectively.

increased in the order by SWPA < viagra < IS aqueous fraction, otherwise in testis tissues, the *e*NOS activity was increased in the order of CON (control, PBS treated) < IS aqueous fraction < SWPA. Overall, the *e*NOS and *n*NOS expression level in the penis increased, and the *n*NOS activity in the testis and brain was suppressed to some extent (Fig. 1 and Fig. 2). This can explain the NO-mediated stimulatory effect of the DMA derived fraction, which might act via the up-regulation of $Ca^{2+}$- dependent NOS expression (Park *et al*., 2004). Furthermore, the *e*NOS activity in the brain tissues of 9 month old rat model for 4 days, *e*NOS activity in testis somewhat suppressed (data not shown).

**Calcium ion assay in sample treated rat.** The increase in $Ca^{2+}$-dependent penile NOS expression can be related the erectile responses and the hemeostatic balance by activating the neuronal and vascular systems (Park *et al*., 2004). The concentration of calcium ions in the serum did not increased in silkworm pupae ethanol extract and *Isaria sinclairii* methanol extract, contrast to high significant increase in viagra treated rat group compared to those of phosphate buffered saline treated control rats (Fig. 5). Also, the concentration of calicium ions in penile tissue lysates did not increased compared to control level (data not shown), explaining Nitric oxide (NO) is a potent inhibitor of platelet activation, that inhibits the agonist-induced increase in cytosolic $Ca^{2+}$ concentration (Crane *et al*., 2005).

**Vascular Endothelial Growth Factor (VEGF) related to anti-angiogenesis.** There are some reports that Pro-VGF-derived peptides induce penile erection in male rats (Succu *et al*., 2004), and Nitric oxide

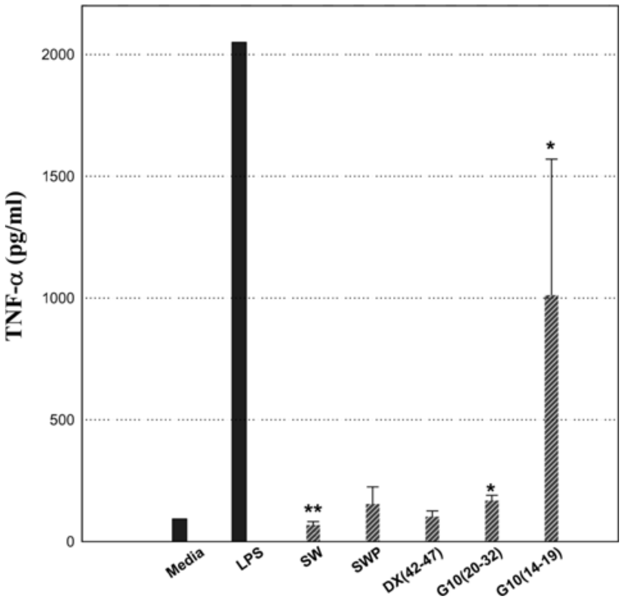

**Fig. 7.** Effects of the silkworm pupae extract on TNF-α production in splenocytes. Silkworm pupae (SWP) DX42-47: 0.4 mg, SWP G10, 20-32: 0.2 mg, SWP G14-19, 0.2 mg; silkworm (SW) ethanol extract 0.8 mg, SWP ethanol extract 0.8 mg. Values represent means ± SD. Significantly different from the untreated controls (*$p < 0.05$; **$p < 0.01$).

enhances angiogenesis via the synthesis of vascular endothelial growth factor and cGMP after stroke in rat (Zhang *et al.*, 2003). In this study, there was little VEGF production in the HUVEC endothelial cells, which suggests that this has virtually no relationship with angiogenesis associated with the cancer progenesis state (Fig. 6). Otherwise, the level of VEGF production in splenocytes did not increase after a 24 hr treatment (data not shown).

**Tumor necrosis factor-α production measurements in splenocytes.** Silkworm pupae partial purification fraction with dimethyladenosine-arabinose, did not increase the level of TNF-α (Fig. 7), showing no relation to inflammation.
